# Supplementary material for: Stretch-Induced Tenomodulin Expression Promotes Tenocyte Migration via F-Actin and Chromatin Remodeling
Source: Int J Mol Sci. 2021 May 6;22(9):4928. doi: 10.3390/ijms22094928 (PMC8124537; doi:10.3390/ijms22094928)
Supplement: Supplementary file 1 [file ijms-22-04928-s001.zip › ijms-1137271-supplementary.pdf]

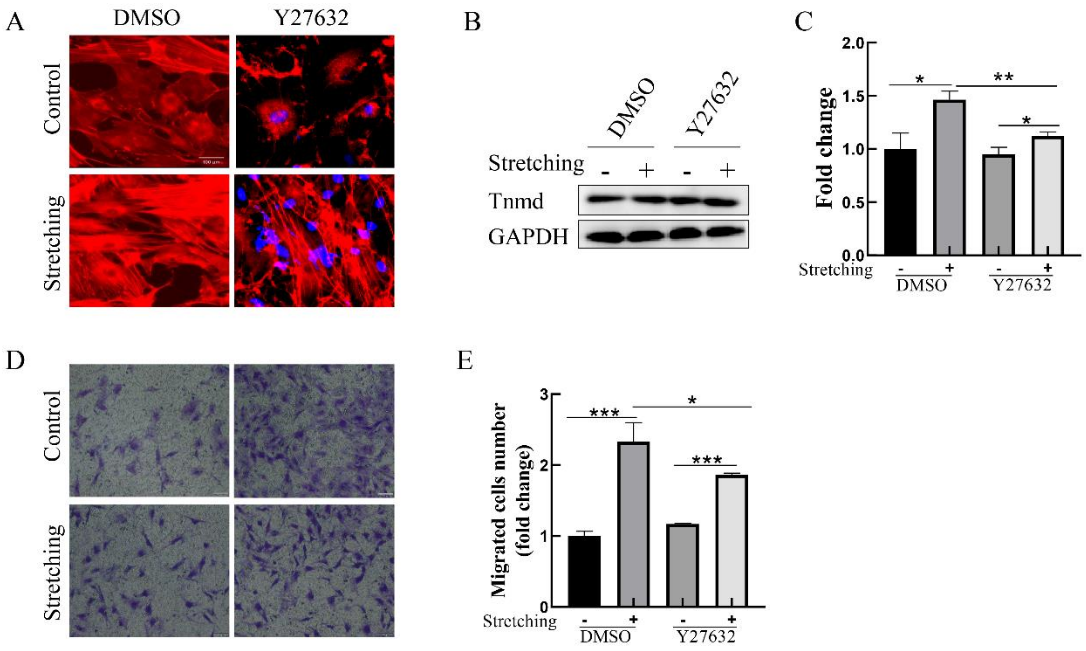

Figure S1. Effect of actin stress fibers inhibitor on stretching-increased Tnmd expression and tenocyte migration. (A) The F-actin organization was detected via phalloidin staining (scale bar, 100 μm). (B) Western blotting was used to analyze the expression of Tnmd protein. (C) Quantification of the Tnmd expression. (D) Migratory tenocytes were detected by a Transwell assay (scar bar, 100 μm). (E) The migration was counted and quantized. The graph shows the mean with SD; $n = 3$, * $p < 0.05$; ** $p < 0.01$; *** $p < 0.001$.

Table 1. The list of primers

Target genes	Name	Primer sequences (5′-3′)
**Tnmd**	Forward	GTGGTCCCACAAGTGAAGGT
	Reverse	GTCTTCCTCGCTTGCTTGTC
**GAPDH**	Forward	GCAAGTTCAACGGCACAGTCA
	Reverse	CACCCCATTTGATGTTAGCGG

Table 2. The list of shRNA

Target genes	Name	sequences (5′-3′)
**Tnmd**	Tnmd-shRNA1 sense	CCGGCGGATACACTGGCATCTACTT
		CTCGAGAAGTAGATGCCAGTGTATCCGTTTTTTG
	Tnmd-shRNA1 antisense	AATTCAAAAACGGATACACTGGCAT
		CTACTTCTCGAGAAGTAGATGCCAGTGTATCCG
	Tnmd-shRNA2 sense	CCGGGAGAGGTTACTGTTGTATTTACT
		CGAGTAAATACAACAGTAACCTCTCTTTTTG

	Tnmd-shRNA2 antisense	AATTCAAAAAGAGAGGTTACTGTTGTA
		TTTACTCGAGTAAATACAACAGTAACCTCTC
**Control**	control-shRNA sense	CCGGCCTAAGGTTAAGTCGCCCTCGCTCG
		AGCGAGGGCGACTTAACCTTAGGTTTTTG
	control-shRNA antisense	AATTCAAAAACCTAAGGTTAAGTCGCCC
		TCGCTCGAGCGAGGGCGACTTAACCTTAGG
